# Controlling Welding Residual Stress and Distortion of High-Strength Aluminum Alloy Thin Plates by a Trailing Hybrid High-Speed Gas Fluid Field

**DOI:** 10.3390/ma15186451

**Published:** 2022-09-16

**Authors:** Guangtao Zhou, Biao Liu, Wei Song, Huachen Li, Jingzhen Kuang, Mingwang Qiu

**Affiliations:** 1 Key Laboratory of Special Energy Field Manufacturing in Fujian Province, College of Mechanical Engineering and Automation, Huaqiao University, Xiamen 361021, China; 2 State Key Laboratory of Advanced Welding and Joining, Harbin Institute of Technology, Harbin 150001, China; 3 School of Mechanical & Electrical Engineering, Xuzhou University of Technology, Xuzhou 221018, China

**Keywords:** trailing hybrid high-speed gas fluid field, aerodynamic load, residual stress, deflection distortion, finite element analysis

## Abstract

This paper presents an investigation of the welding residual stress and distortion of LY12 high-strength aluminum alloy (6061) by improving the local welding thermal and mechanical fields. A trailing hybrid high-speed gas fluid method was proposed and applied to decrease the welding residual stress and distortion of 6061 aluminum alloy efficiently. Firstly, the temperature and stress fields were calculated using the finite element simulation method, considering a trailing hybrid high-speed gas fluid field. The distance between the aerodynamic load and the heat source action was a key factor determined by the simulation method. In addition, the reasonable effective range of gas pressure was obtained. Subsequently, welding and distortion tests were conducted on the self-developed device under conventional welding and high-speed gas fluid field conditions. The results showed that an aerodynamic load under 30 MPa of gas pressure was available near the area at a distance of 20–28 mm from the heat source for thin plate welding distortion. The peak longitudinal residual tensile stresses in the weld’s mid-length section decreased by 77.73%, the peak residual compressive stresses decreased by 69.23% compared with conventional welding, and the deflection distortion disappeared almost entirely. The maximum deflection of the distortion was only 1.79 mm, which was 83.76% lower than the 11.02 mm of the conventional welding distortion. This validates that the method can simultaneously and greatly eliminate the welding residual stress and distortion.

## 1. Introduction

In the context of lightweight materials becoming increasingly mainstream, sheet metal structures are widely used in ships, machinery, chemicals, aerospace, and other fields. However, in general, warpage instability distortion readily occurs in thin plate welding, showing wave-shaped characteristics [1,2]. Welding distortion will seriously affect the accuracy of the dimensions and flatness of the sheet metal structure, making it a huge potential risk in the service process in areas with high safety requirements. Therefore, most sheet metal structures need to be straightened due to the welding distortion, which will increase the manufacturing cycle and production costs, especially for LY12 high-strength aluminum alloy (6061), which has a significant coefficient of linear expansion, leading to a larger distortion after welding. Thus, it is very critical to control its welding distortion.

Traditionally, controlling welding distortion is referred to as post-welding straightening, which requires a great amount of energy; therefore, it is limited in its application in industrial production [3,4,5,6,7,8]. Distortion control during the welding process can improve production efficiency; however, the current mechanism for controlling the weld distortion of high-strength aluminum alloys during the welding process is mainly based on the mechanical effects produced by the energy field. Moreover, the mechanism mainly generates tensile strain in the sheet to counterbalance the compressive strain on the weld metal. Controlling methods commonly used include the mechanical field, thermal field, electromagnetic field, and other energy fields [9,10,11,12,13,14,15]. In recent years, several new techniques have been developed for controlling and improving the welding distortion of thin plates such as adding oxide fluxes or nanoparticles to assist in controlling welding distortion during welding [16,17,18]. Nevertheless, most of the methods make direct contact with the weld zone, thus resulting in indentations, marks, and other notch effects on the weld surface. LY12 high-strength aluminum alloy has high requirements for the surface of the component, and no chipping effect should occur.

Therefore, a new method, trailing hybrid high-speed gas fluid field, during the welding process was proposed to eliminate destabilizing distortion of thin plate welding. A concentrated high-speed gas flow was applied at a distance behind the heat source and remained with the heat source. The aerodynamic loading acted mechanically on the fusion metal that had just started to solidify and shrink; thus, the internal stresses was adjusted, and the weld distortion could be reduced for the welded plate. This method can avoid the severe weakness of post-weld distortion correction for high-strength aluminum alloys and the early failure of the components with sharp notch effects on the surface of weldments caused by conventional methods. It is based on the principle of correcting the aerodynamic load generated by the high-speed gas fluid acting on the weld condensation metal, which causes an additional tensile strain. First, this work established the principal model for controlling the welding distortion of thin plates by welding the high-speed gas fluid field. Then, it compared and analyzed the temperature field, stress field, and distortion law under the condition of a high-speed gas fluid field between conventional welding and follow-on welding. The double-ellipsoidal heat source equation was modified, and the secondary development of the Marc software aerodynamic load subroutine was completed. The residual stresses and flexural distortion of the welded parts were tested under two conditions of aluminum alloy sheet welding, which were in good agreement with the simulation results and verified the correctness of the thermal coupling model of the welding with trailing of the hybrid high-speed gas fluid field.

## 2. Modeling of the Welding of the Trailing of the Hybrid High-Speed Gas Fluid Field

### 2.1. Theoretical Fundamentals

**Figure 1:**
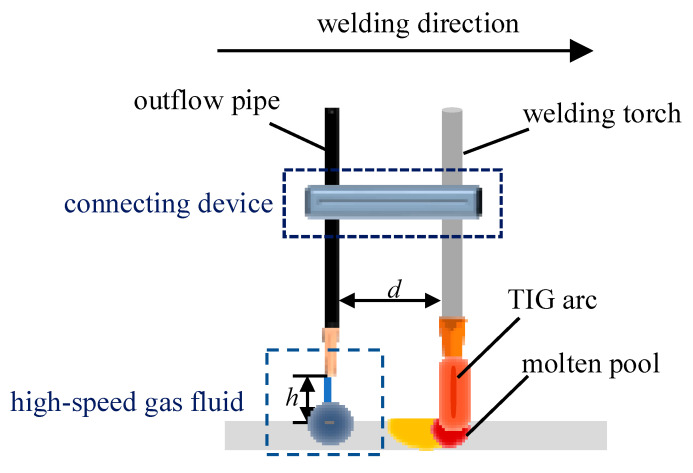
Schematic diagram of the stress and distortion controlled by the trailing of the hybrid high-speed gas fluid field during welding.

A model of welding using the trailing hybrid high-speed gas fluid field for thin plate welding distortion control is shown in Figure 1. The high-speed gas fluid field was arranged at a certain distance behind the welding torch and kept in synchronous motion with the torch by means of a connecting device. The yellow oval part is the low-yield zone after welding, the blue circular area is where the aerodynamic load acted, and the red circular area is the molten pool. The *d* in the figure represents the distance between the aerodynamic load and the heat source, and *h* is the gas outlet height of the aerodynamic load from the workpiece.

### 2.2. The Principle of Controlling the Distortion of Thin Plate Welding with High-Speed Welding

The welding process is a local transient inhomogeneous heating process [18,19,20]. When the weld is heated by a heat source, the metal in the area where the heat source acts is instantaneously heated to the melting point and forms a molten pool. However, the other solid areas do not reach the melting point, and the area adjacent to the molten pool expands by heat [21,22,23,24,25,26]. However, the metal in its vicinity is still in a colder state; thus, the molten pool expands due to the heat, and the metal is compressed and deformed by the action of the metal in its vicinity. When the heat source leaves the region and the molten pool begins to solidify, the metal in the region should contract, but it is restrained in its contraction process by the action of its nearby metal. At this time, the metal in this area is deformed in a tensile plastic manner, and after complete cooling, residual stresses are generated inside the plate. Therefore, the compressive strain generated during heating of the heat source is reduced, or the tensile strain generated during the cooling and solidification phase should increase [27,28,29,30,31]. In addition, when the residual compressive stress inside the plate is less than the critical instability stress of the plate, the purpose of eliminating the instability distortion can be achieved.

Based on this principle, Japanese scholars proposed the theory of intrinsic strain [32], which is expressed as:

(1) $$\left[\varepsilon *\right]=\left[\varepsilon_{Y}\right]+\left[\varepsilon_{L}\right]$$

where [ε*] is the intrinsic strain, [*ε_Y_*] is the compressive strain, and [*ε_L_*] is the tensile strain. That is, the intrinsic strain is composed of the compressive strain generated during the heating of the heat source and the tensile strain generated during the cooling and solidification process. During heating of the heat source, [*ε_L_*] is much smaller than [*ε_Y_*]; thus, [ε*] is always negative. As long as [ε*] is not 0, residual stresses are generated. According to inherent strain theory, the purpose of controlling the welding stress and distortion is to decrease the inherent strain. The tensile strain in the weld area under a high-speed gas fluid loading consists of two components. On the one hand, there is the tensile strain due to the aerodynamic loading. On the other hand, there is the tensile strain influenced by shrinkage during the cooling of the weld. Both effects counteract the compressive strain generated by the thermal expansion of the weld metal. The ultimate goal is to reduce the inherent strain of the thin plate structure and control the welding distortion of the thin plate.

### 2.3. Parametric Characterization of the Aerodynamic Load

According to aerodynamics, high-speed gas fluid can produce mechanical effects [33]. When the distance between the high-speed gas fluid and the workpiece is small, the high-speed gas fluid will produce a stable laminar flow, which can be regarded as an ideal fluid, and it will produce a concentrated force on the surface of the workpiece. According to the theoretical basis of aeroelasticity [34,35], the relationship between the size of the aerodynamic load, F, and other parameters can be deduced, and its expression is as follows:

(2) $$F=\frac{\rho }{2\pi^{2}r^{4}}{\left(\frac{P_{1}}{P_{2}}\right)}^{2}{\left(\frac{V_{1}T_{2}}{T_{1}}\right)}^{2}$$

where *P*_1_ and *P*_2_ are the gas pressures at the inlet and outlet of the outflow pipe, respectively; *T*_1_ and *T*_2_ are the gas temperatures at the inlet and outlet of the outflow pipe, respectively; *V*_1_ is the gas flow rate at the inlet of the outflow pipe; *ρ* is the gas density; *r* is the radius of the outlet of the outflow pipe.

**Figure 2:**
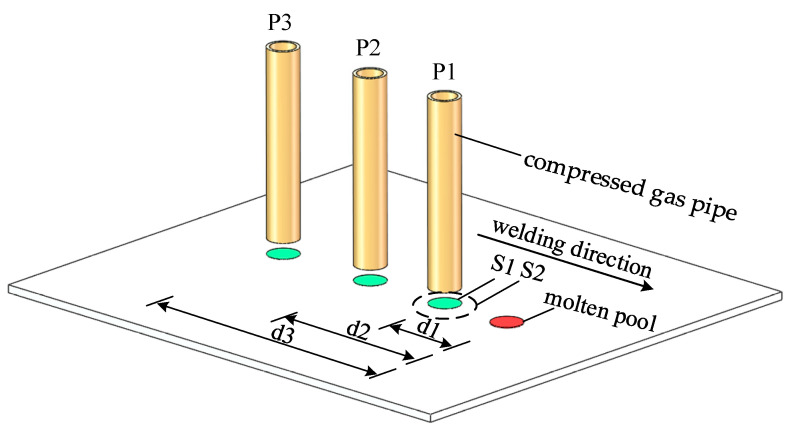
Principal model of the aerodynamic load application.

There is a range of loading distances behind the heat source, and when the loading distance exceeds this range of action, the mechanical effect of the aerodynamic load will not be able to regulate the residual stresses generated by the weld. As shown in Figure 2, the force applied to the weld area by the high-velocity gas behind the heat source can be characterized by the gas pressure: P1, P2, P3, …, Pn; the loading distance: d1, d2, d3, …, dn; the radius of the area of action: S1, S2, S3, …, Sn. The appropriate aerodynamic load can be matched by finetuning the values of each parameter.

## 3. Numerical Simulation of Welding with the Trailing of the Hybrid High-Speed Gas Fluid Field

### 3.1. Finite Element Modeling

**Figure 3:**
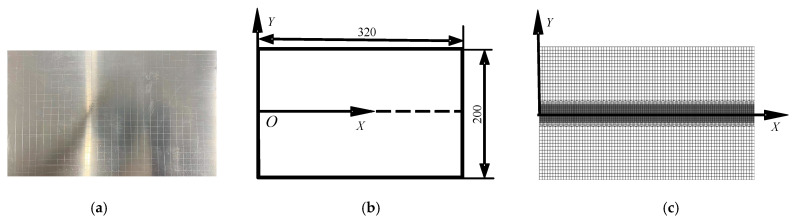
Welding model diagram of an aluminum alloy sheet: (**a**) specimen; (**b**) geometric model; (**c**) finite element model.

Numerical finite element calculation of TIG welding test was carried out using an LY12 aluminum alloy sheet, as shown in Figure 3a. In order to accurately measure the deflection, grids 10 × 10 mm were carved on the specimen. The model size was 320 × 200 × 1 mm, as shown in Figure 3b, and the element type was an eight-node hexahedral element with 48,477 nodes and 32,000 individual elements. In order to ensure the accuracy of the calculation and improve the efficiency of the calculation, the overall number of computational meshes was reduced in the form of sparse transition, and only the critical areas of the weld and aerodynamic loading locations were meshed refined, as shown in Figure 3c.

As shown in Table 1, the material parameters (modulus of elasticity (*E*), linear expansion parameter (*α*), yield limit (*σ_s_*), specific heat (*c*), thermal conductivity (*K*), etc.) of the LY12 aluminum alloy sheet were dependent on the temperature, while the material’s Poisson ratio (*μ*) and mass density (*ρ*) were constant values.

**Figure 4:**
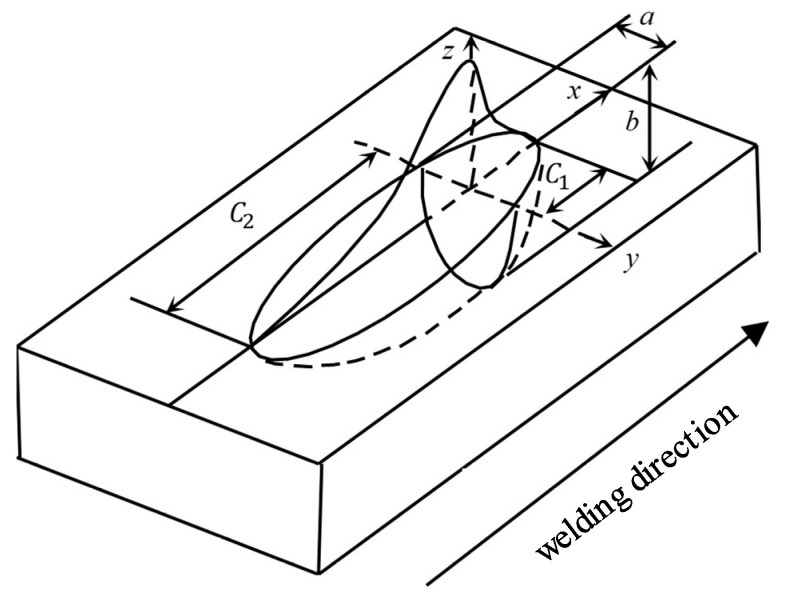
Modified model of the double-ellipsoid heat source. *a* is the half-width of the heat source, *b* is the depth of the heat source, *C*_1_ is the length of the front quadrant of the heat source, and *C*_2_ is the length of the rear quadrant of the heat source.

The welding heat source in this study was modeled using a double-elliptical heat source [37], as shown in Figure 4. The temperature field of the second half of the arc was affected due to the accelerated cooling effect of the high-speed gas fluid behind the arc during the welding process. However, the effect on the temperature field of the front half of the arc was very small; therefore, a modified double-ellipsoidal heat source model was chosen, with the specific expressions as shown in Equations (3)–(5).

The first half of the ellipsoidal heat source distribution is expressed as:

(3) $$q(x,y,z,t)=\frac{6\sqrt{3}f_{1}Q}{abc_{1}\pi \sqrt{\pi }}\exp-3\left(\frac{x^{2}}{a^{2}}+\frac{y^{2}}{b^{2}}+\frac{{\left(z-vt\right)}^{2}}{c_{1}^{2}}\right)$$

The latter half of the ellipsoidal heat source distribution is expressed as:

(4) $$q(x,y,z,t)=\frac{6\sqrt{3}f_{2}Q}{ab\lambda c_{2}\pi \sqrt{\pi }}\exp-3\left(\frac{x^{2}}{a^{2}}+\frac{y^{2}}{b^{2}}+\frac{{\left(z-vt\right)}^{2}}{c_{2}^{2}}\right)$$

The total heat flow density is:

(5) $$q\left(x,y,z\right)=q_{1}+q_{2}$$

where *Q* is the total power of the welding mobile heat source; *f*_1_ and *f*_2_ represent the ellipsoidal heat distribution functions of the front and rear parts, and *f*_1_ + *f*_2_ = 2; *a*, *b*, and *c* are the ellipsoidal shape parameters; *λ* is the heat source model correction factor, which is related to the flow rate of the high-speed gas and heat dissipation coefficient.

### 3.2. Development and Compilation of the Aerodynamic Load Subroutines

Since the aerodynamic load’s impact was not an inherent boundary condition of the Marc software, a secondary development of the software was performed in order to enable the application of this boundary condition. The compilation of the moving load subroutine was completed with Fortran77 as the compilation language, and the impact effect of the aerodynamic load on the LY12 aluminum alloy sheet was realized.

### 3.3. Temperature Field with the Action of Welding with the Trailing of the Hybrid High-Speed Gas Fluid Field

**Figure 5:**
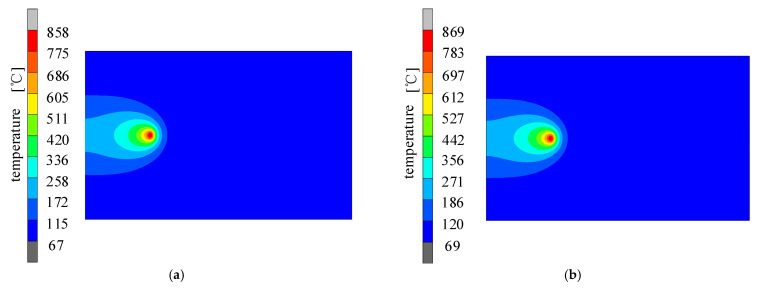

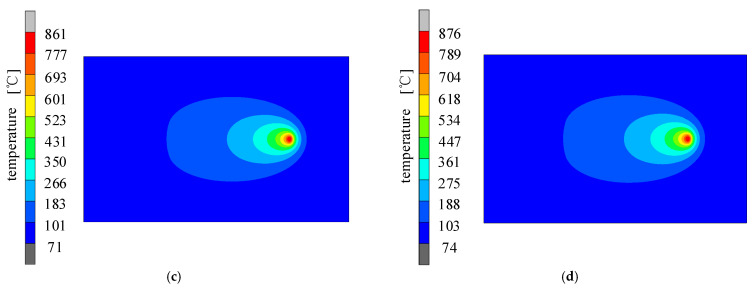
Contours of the temperature field distribution under different conditions: (**a**) under high-speed gas fluid conditions at *t* = 20 s; (**b**) under conventional welding conditions at *t* = 20 s; (**c**) under a high-speed gas fluid condition at *t* = 60 s; (**d**) under conventional welding conditions at *t* = 60 s.

Under the same welding specification parameters, the welding temperature field was simulated and calculated for both conventional welding and welding with a trailing high-speed gas flow. For the latter, the distance of the center of action of the aerodynamic load from the heat source was the key parameter, which was initially set to 12 mm. The calculated contour distribution of the welding temperature field at different moments under both conditions are shown in Figure 5.

As can be seen from the figure, when the welding was at *t* = 20 s and *t* = 60 s, the temperature field of the welding under trailing hybrid high-speed gas fluid conditions was slightly different from conventional welding conditions, and the temperature field error was less than 2% and not significant. The forced convection effect generated by the high-speed gas fluid field on the temperature field of the high-strength aluminum alloy sheet was small; thus, the slight change in the temperature field with the trailing hybrid high-speed gas fluid almost did not affect the welding stress field.

### 3.4. Determination of the Aerodynamic Load Action Distance Range

**Figure 6:**
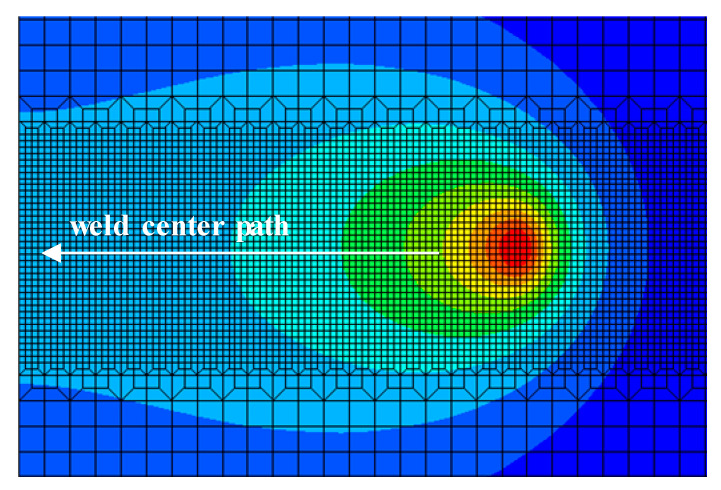
Node temperature sampling path.

The distance of the aerodynamic load from the heat source should be based on the yield strength of the base or weld metal at different locations from the heat source at the weld centerline, and the yield strength of the material is related to the temperature. The nodal temperatures of the weld metal at different distances from the heat source were collected in the path followed by the direction shown in Figure 6. 

**Figure 7:**
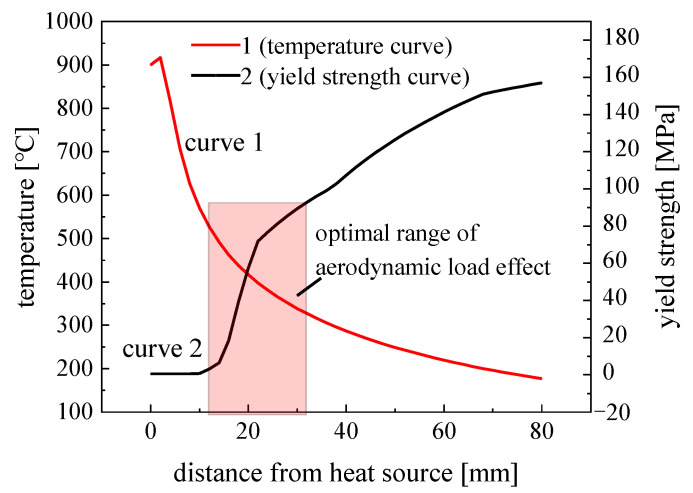
Relationship between temperature and yield strength at different positions from the arc.

In order to analyze the relationship between the temperature and yield strength at different positions, the two curves were combined for comparison. The nodal temperatures of the weld metal at different distances from the heat source are shown in Figure 7 (curve 1), and the yield strength of the material corresponding to the node temperature at different distances from the heat source was obtained according to the thermophysical property parameters of the material, as shown in Figure 7 (curve 2). As can be seen from Figure 7, the farther the distance from the heat source, the lower the node temperature. Within 12 mm from the heat source, the node temperature decreased faster, and when the distance from the heat source was greater than 12 mm, the node temperature decreased more slowly. The farther the distance from the heat source, the lower the temperature of the node, thus the higher the yield strength of the material. Within 12 mm from the center of the heat source, the yield strength of the material was only within 1 MPa. Ranging from 12 to 24 mm, the yield strength value of the material increased sharply. When the distance from the heat source was greater than 24 mm, the yield strength of the material steadily increased, and by 32 mm, the yield strength reached over 100 MPa.

According to the relationship between the distance from the heat source and the yield strength, this experiment could initially determine the range of the aerodynamic load action distance. When the material yield strength fluctuated within only 1 MPa, the material in this region was close to the “mechanical melting point”, and unrecoverable plastic distortion easily took place. However, when the yield strength of the material reached 100 MPa or more, an aerodynamic load device was required to provide a high level of energy, which is difficult to perform. Therefore, the initial determination of the aerodynamic load action distance was 12–32 mm, as shown in the rectangular shadow area in Figure 7. In order to explore the action law of the aerodynamic load and reduce the number of simulation calculations, distances from the heat source of 12, 16, 20, 24, 28, and 32 mm were selected as the positions of action of the aerodynamic load. Table 2 lists the calculated range of the aerodynamic load meter at different loading distances.

### 3.5. Variations in the Welding Residual Stress under an Aerodynamic Load

**Figure 8:**
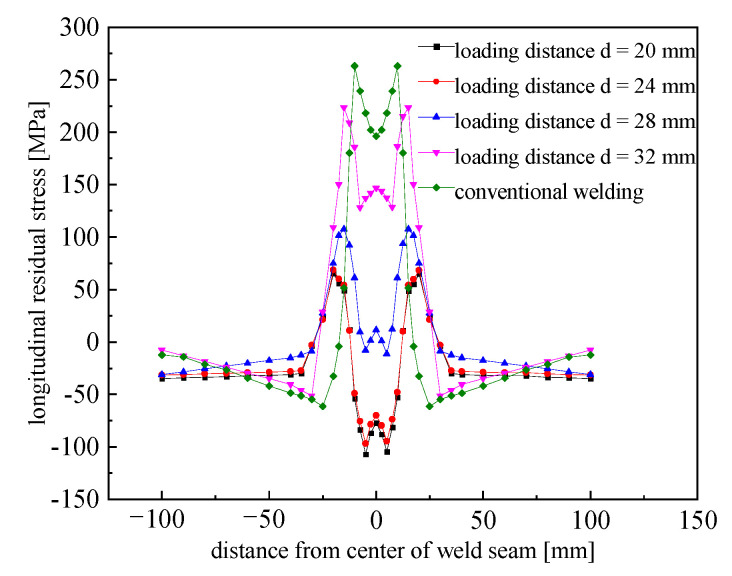
Comparison of the longitudinal residual stress in the middle section under different conditions.

In order to analyze the effect of the different loading distances on the longitudinal residual stress, the longitudinal residual stress values in the mid-length section at different loading distances were extracted from the finite element simulation results for comparison with conventional welding conditions. For an aerodynamic load of 30 MPa and loading distances (*d*) of 20, 24, 28, and 32 mm, the longitudinal residual stress results are shown in Figure 8.

As can be seen from the figure, welding with high-speed gas fluid field conditions for the control of residual stresses in the weld was much more effective. In particular, in the weld zone, which is the area of direct action of the aerodynamic load, the residual stress decreased much more significantly. When the loading distances (*d*) were 20, 24, 28, and 32 mm, the stresses in the center of the weld were −70.34, −69.87, 11.47, and 146.66 MPa, respectively. The peak tensile stress decreased by 74.90%, 73.76%, 58.93%, and 15.01%, respectively, compared with conventional welding. Under the loading distances (*d*) of 20 and 24 mm, the residual stress at the center of the weld converted from tensile stress to compressive stress.

As the loading distance increased, the longitudinal residual stress had a similar distribution to that under conventional welding conditions. The peak tensile stress and peak compressive stress also increased with the increases in the loading distance, and the area of tensile stress on the specimen gradually decreased. This was due to the fact that when the loading distance increases, the temperature of the metal in the area of action decreases and the yield strength increases. However, the aerodynamic load was kept constant at 30 MPa, and the plastic ductile effect on the metal in the area of action was reduced. Therefore, the controlling effect of the longitudinal residual stress was reduced. When *d* = 20 and 24 mm, the aerodynamic load of 30 MPa was greater than the yield strength of the material in the area of action. At this time, plastic distortion of the metal in area of action occurred, and the elastic distortion recovery of the surrounding metal was limited. As a result, the stress inside the plate could no longer be further changed; thus, the differences in the longitudinal residual stress under *d* = 20 mm and *d* = 24 mm were smaller.

### 3.6. Analysis of the Welding Deflection Distortion under an Aerodynamic Load

**Figure 9:**
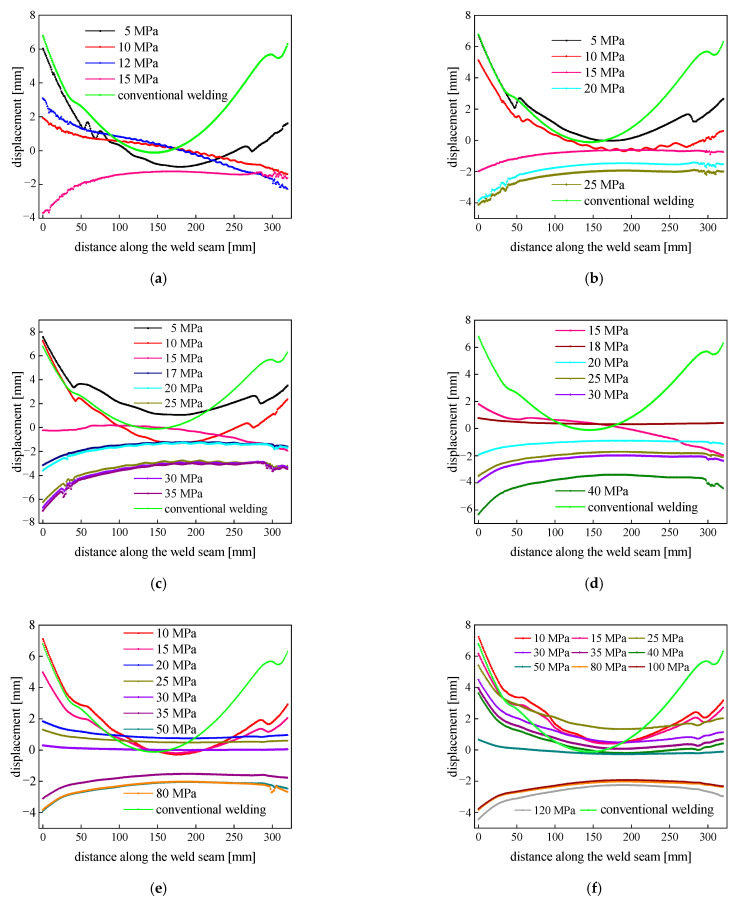
Distortion diagram of thin plates under different loading distances: (**a**) *d* = 12; (**b**) *d* = 16; (**c**) *d* = 20; (**d**) *d* = 24; (**e**) *d* = 28; (**f**) *d* = 32 mm.

The welding distortion of thin plates at different loading distances (*d*) is shown in Figure 9. As can be seen from the plot, when the loading distance (*d*) was less than 20 mm, the required aerodynamic load was small enough to control the distortion of the LY12 aluminum alloy sheet, but the controlling effect was not more evident. When the loading distance was greater than 32 mm, the aerodynamic load required to control the distortion of the LY12 aluminum alloy sheet was too large. Thus, when the loading distance was between 24 and 32 mm, the applied aerodynamic load could effectively control the welding distortion of the LY12 aluminum alloy thin plates. In addition, when the loading distance was 28 mm and the aerodynamic load was 30 MPa, the controlling effect was much better, and the weld displacement decreased by 94.20% compared with conventional welding.

## 4. Welding Test with the Trailing of the Hybrid High-Speed Gas Fluid Field

### 4.1. Welding Test

**Figure 10:**
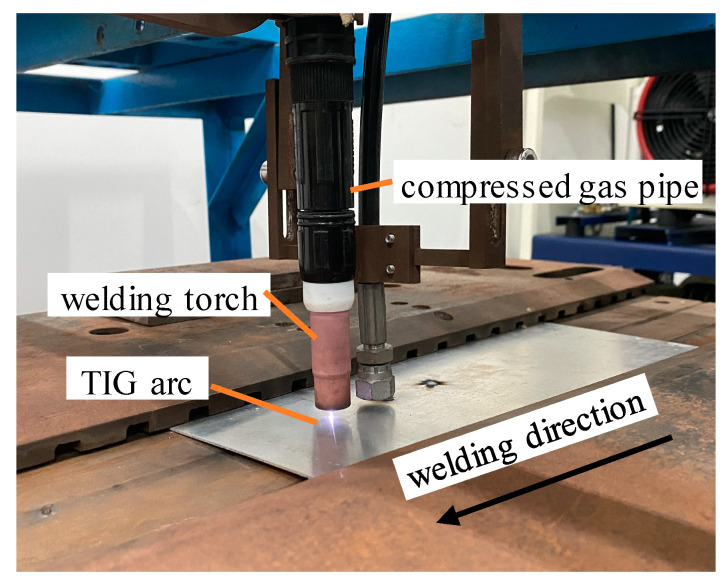
Image of the high-speed gas fluid field device used during the welding process.

In order to verify the validity of the simulation results, the test materials and dimensions of the specimen were the same as those of the finite element calculation model. Moreover, Table 3 lists the chemical composition of the materials, and the test apparatus consisted of a welding torch and a compressed gas pipe, as shown in Figure 10. AC TIG welding was selected for the test, and the welding was conducted in the form of surface deposition on the thin plates. Full penetration was achieved due to the thin dimensions of the thickness. The welding machine model was a Panasonic YC-300WX4, and the welding parameters are listed in Table 4.

The aerodynamic load generator model was a PGH30-0.1XT with an adjustable gas pressure range from 0.1 to 60 MPa. Nitrogen of 99.99% purity was used as the test compressed gas, and argon of 99.99% purity was applied as the welding shielding gas.

**Figure 11:**
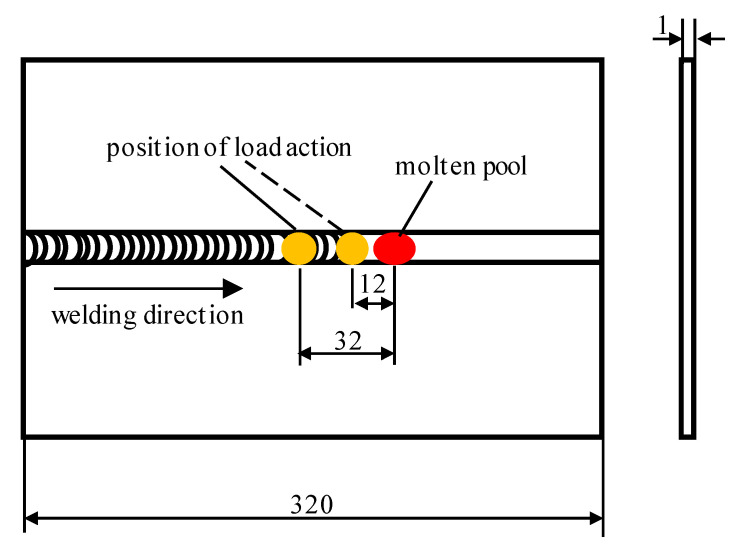
Adjustment range of the loading distance (*d*).

The initial loading distance was 12 mm, and the loading distance was gradually increased in 1 mm increments until it reached 32 mm. Figure 11 shows the relationship between the specimen’s size and the range of the loading distance (*d*). The welding was performed by controlling the pressure output of the oil-lubricated compressor to obtain different gas pressures. The test results showed that the specimen was well formed, and the distortion was well straightened when the loading distance (*d*) was 20 mm and the gas pressure was 30 MPa.

### 4.2. Longitudinal Residual Stress Test under a High Speed Gas Fluid Field during Welding

**Figure 12:**
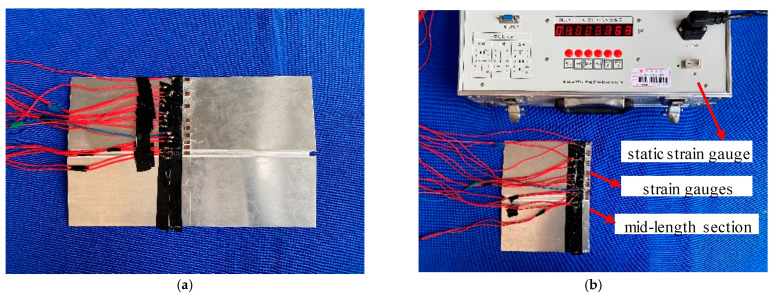
Test images of the strip cutting method: (**a**) before cutting; (**b**) after cutting.

**Figure 13:**
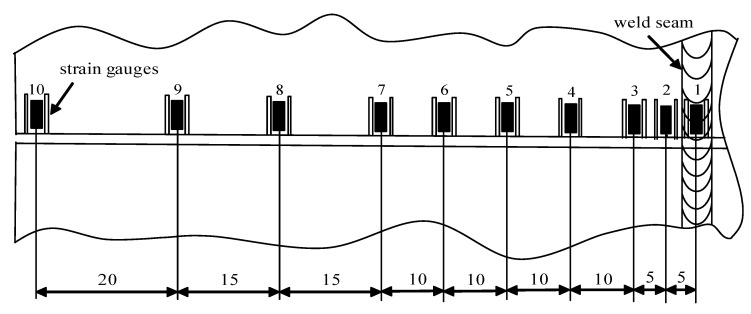
Strain gauge location arrangement for stress measurement.

The residual stress test was conducted using the strip-cutting method, as shown in Figure 12. It was carried using the static strain gauge model CM-1A-20 to measure the stress of the welded specimen’s mid-length section under conditions of conventional welding and a high-speed gas flow field (*d* = 20 mm, *P* = 30 MPa). Since the residual stresses on both sides of the weld were symmetrically distributed along the weld’s centerline, it was sufficient to mount strain gauges on only one side of the welded specimen. Gauges were spaced close together near the weld where the residual stresses varied drastically, while the stress in the farther area varied slowly. The strain gauges were arranged schematically, as shown in Figure 13. Strips approximately correspondingly wide were cut from the specimen.

**Figure 14:**
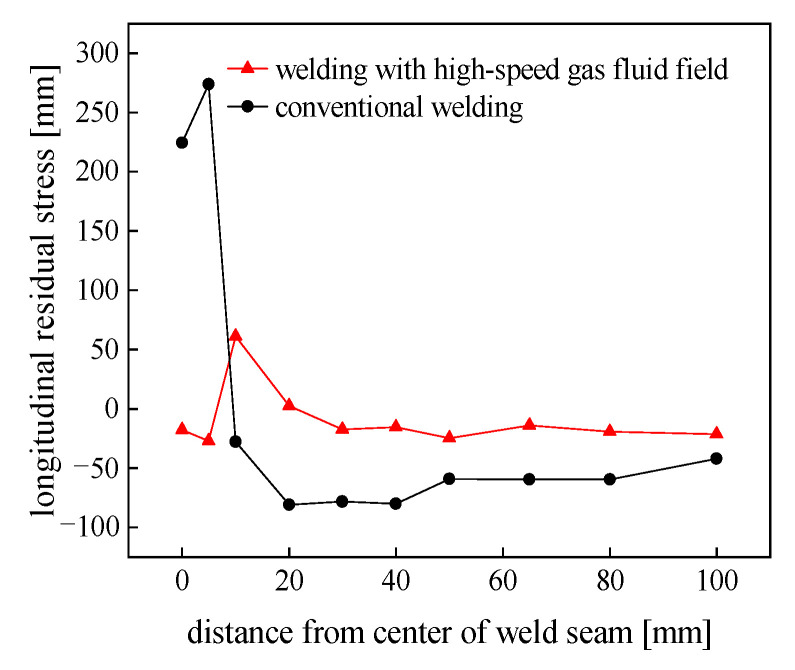
Comparison of the longitudinal residual stress.

The residual stresses presented in the welded specimen were released by cutting the welded specimen along the mid-length section and then cutting both sides of the strain gauge along its long edge direction. The change in the elastic strain caused by the release of stress was indicated by a static strain device, and then the longitudinal residual stress was calculated using Hooke’s law. The longitudinal residual distribution of the welded specimens under different conditions can be seen in Figure 14. Note that with the application of a trailing aerodynamic load during welding, the peak tensile stress had a larger reduction. The overall residual stress obviously decreased compared to that of conventional welding. Among them, the peak tensile residual stress location of the trailing aerodynamic load was different from that of conventional welding, slightly far from the center of the weld. Moreover, the longitudinal residual stress at the center of the weld and in the vicinity of the weld area significantly decreased. All the test results were in excellent agreement with the results of the FEM numerical simulation, which illustrates the accuracy of the above numerical simulation results. This was due to the fact that sufficient stretching was caused to the metal in the area of action by the application of the aerodynamic load, effectively counterbalancing the longitudinal compressive plastic distortion produced during the welding process. Furthermore, the inherent strain of the metal in the welded joint area was reduced, resulting in lower residual stresses in the sample. According to the results of the comparison, the maximum tensile residual stress under a high-speed gas flow field condition was 61.02 MPa, which decreased by 77.73% over that of conventional welding, and the peak residual compressive stress was −24.87 MPa, which decreased by 69.23%.

### 4.3. Flexural Distortion Test under a High-Speed Gas Fluid Field during Welding

**Figure 15:**
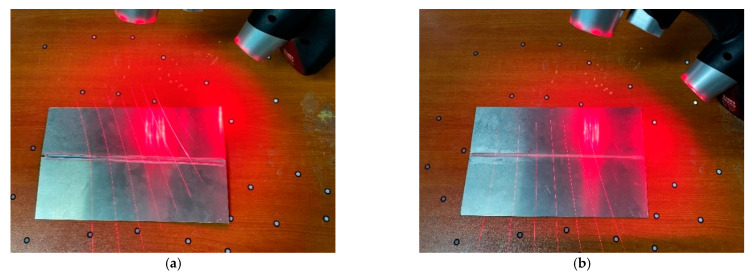
Laser scanning of an LY12 aluminum alloy welding specimen under different conditions: (**a**) conventional welding; (**b**) welding with a trailing hybrid high-speed gas fluid field.

**Figure 16:**
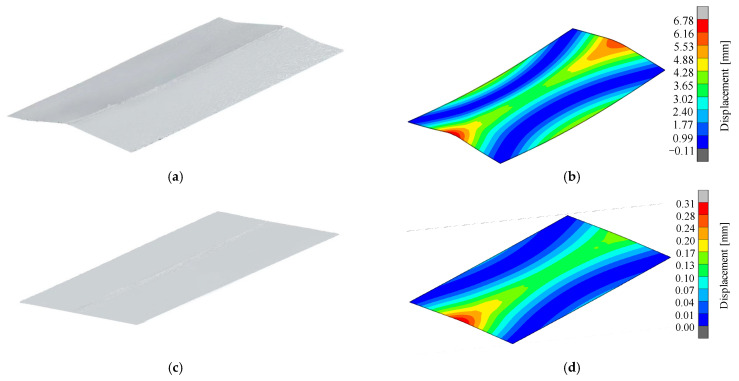
Comparison between the scanned form of the diagram of the sheet distortion and the numerical simulation results: (**a**) scanning diagram of conventional welding; (**b**) diagram of the simulation results of conventional welding; (**c**) scanning diagram of welding with a trailing hybrid high-speed gas fluid field; (**d**) diagram of the simulation results of welding with a trailing hybrid high-speed gas fluid field.

Due to the large coefficient of linear expansion and small modulus of elasticity for LY12 aluminum alloy, the residual stress took place in the weldment heated unevenly by the heating source, which caused it to easily deform in flexure after welding. In order to verify the ability to control the welding distortion of an LY12 aluminum alloy sheet under high-speed gas fluid field conditions, a comparison was made to analyze the distortion of the LY12 aluminum-alloy-welded specimens under conventional welding and high-speed gas fluid field conditions. As shown in Figure 15, a handheld laser 3D scanner was used to scan the out-of-plate distortion of the welded specimens of the LY12 aluminum alloy under two conditions. The model was an HSCAN771, with an accuracy of 0.01 mm, the test temperature was 20 °C, and the 3D point cloud data regarding the displacement of the specimen were obtained after scanning, as shown in Figure 16a,c.

The laser scanning results under different conditions were compared with the finite element simulation results, as shown in Figure 16. The figure shows that the test results were in good agreement with the finite element simulation results, and the LY12 aluminum alloy conventionally welded specimens deformed in deflection, while almost no distortion occurred during the welding under high-speed gas field conditions. It was found that the maximum distortion occurred along the transverse edge of the aluminum alloy rectangular plate. With a ruler, experiments were conducted to measure the deflection distortion value of the plate’s edge regarding the width of the welded thin plate specimen under different conditions.

**Figure 17:**
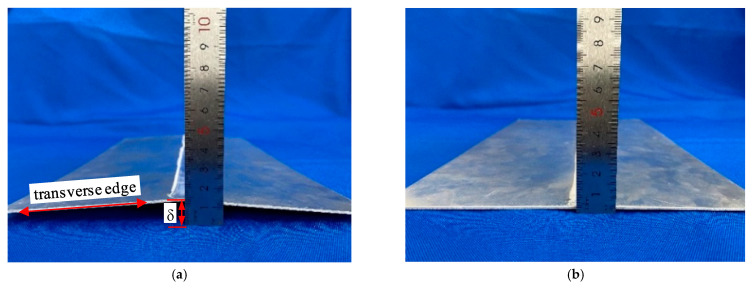
Deflection measurement results: (**a**) conventional welding; (**b**) welding with a trailing hybrid high-speed gas fluid field.

The results showed that the plate edge deflection (δ) of the welded specimen was 11.02 mm under that of the conventional welding conditions, while the deflection was only 1.79 mm under conditions of a trailing high-speed gas fluid field. Compared to conventional welding, the deflection decreased by 83.76%. As shown in Figure 17, the results were consistent with the laser 3D scanning results, further proving that under trailing high-speed gas fluid field conditions, welding distortion can be effectively controlled.

## 5. Conclusions

The influence of welding with a trailing hybrid high-speed gas fluid field on the welding residual stress and distortion of LY12 aluminum alloy was studied. The following conclusions were drawn:

(1) A new method was proposed to control the welding distortion of high-strength aluminum alloy sheet by “welding with a trailing hybrid high-speed gas fluid field”. It was based on the original perspectives of mechanical mechanisms and the concept of “flexible” control. Otherwise, a welding distortion control model was established with a trailing hybrid high-speed gas fluid field, which was also parametrically characterized;
(2) Through the numerical simulations of the temperature field of the welding process under conditions of welding with a trailing hybrid high-speed gas fluid field, the effective action distance (*d*) between the aerodynamic load and the heat source was determined to range from 12 to 32 mm. Various aerodynamic loads were set according to the corresponding loading distance (*d*). The longitudinal residual stresses in the mid-length section were measured at an aerodynamic load loading distance of 20 mm and a gas pressure of 30 MPa;
(3) The aerodynamic load applied to the weld could effectively reduce the residual stress of the weld. The control effect was good when the loading distance was 20–28 mm and the aerodynamic load was 30 MPa. The maximum tensile residual stress under a high-speed gas flow field condition was 61.02 MPa, which decreased by 77.73% over that of conventional welding, and the peak residual compressive stress was −24.87 MPa, which decreased by 69.23%.
(4) The maximum deflection of LY12 aluminum-alloy-welded parts under conditions of welding with a trailing hybrid high-speed gas fluid field was 1.79 mm, which was 83.76% lower than the 11.02 mm of conventional welding.

## Figures and Tables

**Table 1:** Material performance parameters [36].

*T/*°C	*E*/GPa	*α*/(10^−6^ °C^−1^)	*σ_s_*/MPa	*c*/(J·kg^−1^ °C^−1^)	*K*/(W·m^−1^ °C^−1^)
20	70.0	22.8	300	900	117
100	60.8	23.1	280	921	121
200	54.4	24.7	240	1005	126
300	43.1	25.5	160	1047	130
400	32.0	26.5	113	1089	138

**Table 2:** Range of the aerodynamic loads at different loading distances.

Loading Distance (mm)	Aerodynamic Load Range (MPa)
12	5–15
16	5–25
20	5–35
24	10–40
28	10–80
32	10–120

**Table 3:** Chemical compositions of the LY12, wt.%.

Mg	Si	Cu	Cr	Fe	Zn	Mn	Al
0.8	0.4	0.15	0.04	0.7	0.25	0.15	Remaining

**Table 4:** Welding process parameters of automatic TIG welding.

Plate Thickness (mm)	Tungsten Pole Diameter (mm)	Welding Current (A)	Arc Voltage (V)	Welding Speed (mm·s^−1^)	Argon Gas Flow (L/Min)
1	1.6	70	12–15	4	14

## Data Availability

Not applicable.
